# Microbial hitchhikers harbouring antimicrobial-resistance genes in the riverine plastisphere

**DOI:** 10.1186/s40168-023-01662-3

**Published:** 2023-11-01

**Authors:** Vinko Zadjelovic, Robyn J. Wright, Chiara Borsetto, Jeannelle Quartey, Tyler N. Cairns, Morgan G. I. Langille, Elizabeth M. H. Wellington, Joseph A. Christie-Oleza

**Affiliations:** 1 School of Life Sciences, University of Warwick, Coventry, CV4 7AL UK; 2 Present address: Centro de Bioinnovación de Antofagasta (CBIA), Facultad de Ciencias del Mar y Recursos Biológicos, Universidad de Antofagasta, 1271155 Antofagasta, Chile; 3 Department of Pharmacology, Faculty of Medicine, Dalhousie University, Halifax, Canada; 4 Department of Biology, University of the Balearic Islands, 07122 Palma, Spain

## Abstract

**Background:**

The widespread nature of plastic pollution has given rise to wide scientific and social concern regarding the capacity of these materials to serve as vectors for pathogenic bacteria and reservoirs for Antimicrobial Resistance Genes (ARG). *In-* and *ex-situ* incubations were used to characterise the riverine plastisphere taxonomically and functionally in order to determine whether antibiotics within the water influenced the ARG profiles in these microbiomes and how these compared to those on natural surfaces such as wood and their planktonic counterparts.

**Results:**

We show that plastics support a taxonomically distinct microbiome containing potential pathogens and ARGs. While the plastisphere was similar to those biofilms that grew on wood, they were distinct from the surrounding water microbiome. Hence, whilst potential opportunistic pathogens (i.e. *Pseudomonas aeruginosa*, *Acinetobacter* and *Aeromonas*) and ARG subtypes (i.e. those that confer resistance to macrolides/lincosamides, rifamycin, sulfonamides, disinfecting agents and glycopeptides) were predominant in all surface-related microbiomes, especially on weathered plastics, a completely different set of potential pathogens (i.e*. Escherichia*, *Salmonella*, *Klebsiella* and *Streptococcus*) and ARGs (i.e. aminoglycosides, tetracycline, aminocoumarin, fluoroquinolones, nitroimidazole, oxazolidinone and fosfomycin) dominated in the planktonic compartment. Our genome-centric analysis allowed the assembly of 215 Metagenome Assembled Genomes (MAGs), linking ARGs and other virulence-related genes to their host. Interestingly, a MAG belonging to *Escherichia* –that clearly predominated in water– harboured more ARGs and virulence factors than any other MAG, emphasising the potential virulent nature of these pathogenic-related groups. Finally, *ex-situ* incubations using environmentally-relevant concentrations of antibiotics increased the prevalence of their corresponding ARGs, but different riverine compartments –including plastispheres– were affected differently by each antibiotic.

**Conclusions:**

Our results provide insights into the capacity of the riverine plastisphere to harbour a distinct set of potentially pathogenic bacteria and function as a reservoir of ARGs. The environmental impact that plastics pose if they act as a reservoir for either pathogenic bacteria or ARGs is aggravated by the persistence of plastics in the environment due to their recalcitrance and buoyancy. Nevertheless, the high similarities with microbiomes growing on natural co-occurring materials and even more worrisome microbiome observed in the surrounding water highlights the urgent need to integrate the analysis of all environmental compartments when assessing risks and exposure to pathogens and ARGs in anthropogenically-impacted ecosystems.

Video Abstract

**Supplementary Information:**

The online version contains supplementary material available at 10.1186/s40168-023-01662-3.

## Background

Plastic litter is a relatively new material that is colonised by a diverse range of microorganisms due to its global ubiquity, e.g. terrestrial, freshwater and marine water bodies, as well as extreme environments [16, 61, 153]. The complexity of such microbial communities has attracted much attention, especially after the term plastisphere was used to define this new ecological niche [160]. Since then, most research efforts have focussed on establishing microbial communities’ temporal and spatial development on plastic debris in marine ecosystems [101, 103, 104, 115]. On the contrary, characterisations of freshwater plastispheres are scarce, despite freshwater bodies (e.g. rivers, streams) being the primary path for plastics’ entry to the ocean [17, 18] as well as a recognised source of potential pathogens [55, 142].

Rivers are estimated to annually transport between 1.15 and 2.41 million tonnes of plastic debris to the oceans [76]. However, a recent estimation has lowered the global plastic waste input from rivers up to three orders of magnitude to 3.5 thousand tonnes [145]. Regardless of the exact amount, once plastic enters a waterbody, its surface is colonised within minutes by the local microbial community [48, 53, 74]. When initially describing the plastisphere, Zettler et al*.* detected a high abundance of *Vibrio* as part of the community colonising polypropylene (PP), which suggested that plastic debris could be a niche for the proliferation and dissemination of opportunistic pathogens [160]. Subsequently, this idea has gained strength given the buoyancy and resilience to environmental degradation of plastics, compared with other naturally occurring surfaces for attachment (e.g. leaves, wood) [5, 29, 71]. Furthermore, antimicrobial resistance (AMR) –mainly studied via the detection of antimicrobial resistance genes (ARGs)– is also prevalent in the plastisphere [57, 143]. AMR is an environmental and public health issue directly linked to the infectious processes of pathogenic bacteria. It has been estimated that AMR-related infections caused 1.27 million deaths worldwide in 2019 (survey including 204 countries and territories) [96], and they are predicted to produce 10 million deaths worldwide by 2050 [100].

The most abundant type of plastic that pollutes the environment is polyolefins, e.g. polyethylene (PE) and PP, matching their dominance in global industrial production (30% and 19.7% of global plastic production for PE and PP, respectively) [117]. Moreover, materials made of PE and PP are less dense than water and therefore float, increasing their transportation within waterbodies [49]. Unsurprisingly, these are the preferred materials when studying microbial colonisation and environmental pathogen occurrence, however, plastic weathering (i.e. polymer oxidation due to abiotic factors) is rarely considered in these studies despite its known influence on the plastisphere [8, 48, 75, 153]. Most commonly, community analyses of the plastisphere are based on the taxonomic results of amplicon sequencing [153], and the potential for microbial pathogenicity is frequently assessed by qPCR [70, 75]. These are both targeted molecular techniques that provide limited information on the microbial community complexity and do not allow investigation of the specific taxa harbouring ARGs or virulence determinants [22, 75]. To date, few investigations have performed comprehensive metagenomic analyses on plastic samples recovered from marine environments [30, 116, 134, 157] and even less from freshwater systems [154]. In this sense, a metagenomic approach provides a more complete description of microbial communities and their genomic plasticity.

In this study, we go beyond a descriptive metagenomic characterisation of *in-situ* riverine plastispheres and complement this approach with controlled *ex-situ* incubations of plastics in freshwater microcosms. Specifically, using our *in-situ* incubations and metagenomic analyses, we characterised the taxonomic profiles of microbial communities colonising both pristine and weathered PE, as well as a control surface (i.e. wood) and the surrounding water. Applying both sequence-driven and Metagenome Assembled Genome (MAG)-driven approaches the metagenomic data allowed a pioneering investigation of the ARGs and virulence factors encoded within these microbial assemblages. Additionally, we performed controlled *ex-situ* incubations to test whether the presence of sub-inhibitory concentrations of antibiotics within the water influence the abundance of ARGs on pristine plastic (PE and PP) and wood. This paper was posted as a preprint on 8th May 2023 and the preprint can be found here https://www.researchsquare.com/article/rs-2886255/v1.

## Results and discussion

### Microbial diversity within riverine plastispheres

**Fig. 1:**
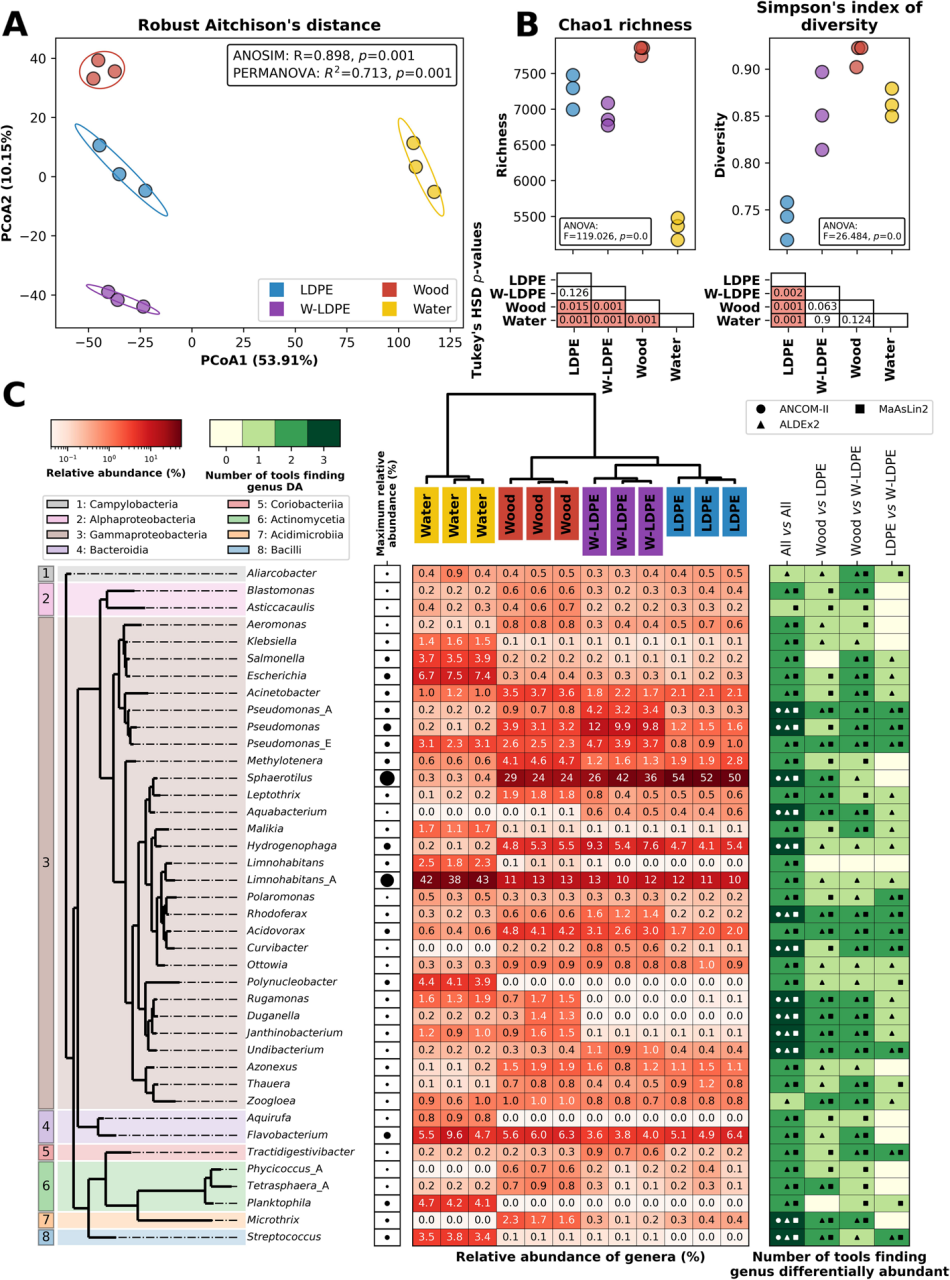
Microbial community analysis of 7-day-old biofilms grown on LDPE, W-LDPE and wood, as well as the riverine planktonic community of the surrounding water. **A** Principal Component Analysis (PCoA) showing samples grouped by Robust Aitchison’s distance (i.e. Euclidean distance of robust Centered-Log Ratio transformed counts). The variation accounted for by each principal component is indicated in parentheses on the axes. Ellipses show the mean of three sample replicates for each treatment plus the standard deviation. ANOSIM and PERMANOVA tests between the treatments are shown in the box. **B** Chao1 richness and Simpson’s diversity index in the three replicates for each treatment. The results of ANOVA tests for differences between treatments are shown in boxes within the axes, while *p*-values for *post-hoc* Tukey’s honestly significant difference (HSD) between treatments are shown underneath (highlighted in red are significant values; *p* ≤ 0.05). Both (**A**) and (**B**) show results for reads classified to the species level. **C** Forty most abundant bacterial genera detected amongst all metagenomes (i.e. those above 0.5% relative abundance). Bacterial genera are grouped by phylogenetic similarity. Colour shading indicates the class each genus belongs to. The relative abundance of each genus (in %) is shown in the central heatmap, normalised *per* column. The top dendrogram shows samples grouped by Robust Aitchison’s distance. The heatmap on the right shows whether taxa were significantly differentially abundant between conditions. We used three tools to determine whether taxa were differentially abundant, ANCOM-II, ALDEx2 and MaAsLin2 (Table S2). White represents that no tool found the genus to be differentially abundant between conditions, while dark green shows that all three tools found a difference. Shapes within cells denote which of the three tools found a significant difference

Pristine and weathered low-density PE films (LDPE and W-LDPE), together with wooden strips as a control surface, were incubated *in-situ* 1 km downstream from the effluent of a wastewater treatment plant (WWTP) in the River Sowe for one week (Coventry, West Midlands, UK; Figs. S1 and S2) after which total DNA was extracted and sequenced (Table S1A). One week was needed for biofilms to establish and allow the development of sufficient biomass for DNA extraction and metagenomic analysis. As expected, a distinct microbial community associated with the materials (i.e. wood, LDPE and W-LDPE) developed compared with the surrounding water (Fig. 1A and Table S1B). Principal Coordinate Analysis (PCoA) showed that all samples clearly clustered by sample type using Robust Aitchison’s distance (PERMANOVA *R* = 0.898, *p* = 0.001; ANOSIM *R*^*2*^ = 0.713, *p* = 0.001; Table S1C), with water being separated from all substrates on the first axis (representing 53.9% variation) and the three substrates being separated on the second axis (10.1% variation; Fig. 1A). Planktonic *vs.* biofilm community differences are well documented [153], and come as a consequence of the different nature of surface-attached *vs.* free-living communities and their capacity to become sessile [24]. Furthermore, water samples represent only a snapshot of the community present at the time of sample collection. In contrast, the substrates represent a cumulative and changing microbial community in the river over the entire incubation period.

Differential microbial assemblages on surfaces have previously been reported between plastic and non-plastic materials [40, 105, 137, 160]. However, the material preferences of microbial colonisers are unclear and may be dependent on residence time, location [101, 104] and nutrient availability [102]. The potential differences across microbial communities colonising plastics rely on the presence of specific early settlers and rare taxa [153], whereas the microbial community differences on wood seem to be shaped by the more degradable nature of this substrate per se [101]. Here it is likely that W-LDPE, as well as wood, also released readily available compounds for microbial biodegradation that caused an early selection of specific taxa, as discussed below. Consistent with previous studies [90], our data showed wood to support the highest alpha diversity values across all samples (ANOVA *p* ≤ 0.05; Fig. 1B and Table S1D). Given the necessity to filter out extremely low abundance taxa during the data analyses, water microbial communities exhibited significantly lower richness (ANOVA *p* ≤ 0.05) compared to all other groups, although the Simpson’s Index of diversity was similar to that of W-LDPE (Fig. 1B). LDPE and W-LDPE were similar in richness to each other, with LDPE having significantly lower Simpson’s Index of diversity than any other sample group (ANOVA *p* ≤ 0.05). The differences in the microbial profiles between the pristine and weathered LDPE are probably related to physicochemical modifications of the weathered material, which reduce the polymer hydrophobicity [11] and promote the release of carbon leachates [126, 159], all being factors that influence microbial settling. Such a phenomenon was also evidenced at the community level during the early colonisation of PE in marine environments [48].

Regarding the affiliation at high taxonomic levels, the vast majority of classified reads were related to Bacteria (98.83% on average within samples), with Eukaryota, Viruses and Archaea making up only 1.01%, 0.15% and 0.01%, respectively (Fig. S3 and Table S1E). Amongst Bacteria, the phylum Proteobacteria dominated across all microenvironments tested (i.e. LDPE, W-LDPE, wood, and water; averaging 90, 93, 85 and 77%, respectively), followed by Bacteroidota (averaging 6, 4, 7 and 8%, respectively; Fig. S3 and Table S1E), similar to a previous metagenomic analysis of water samples at a location nearby to our incubations [28]. Amongst Proteobacteria, the most abundant class was Gammaproteobacteria, averaging > 70% of reads in all samples (Fig. S3), and this was mainly composed of the order Burkholderiales (average 82, 69, 68 and 58%, respectively; Fig. S3). The order Burkholderiales, previously classified as Betaproteobacteria [42], has been reclassified to Gammaproteobacteria in the GTDB taxonomy [111] based on the phylogenetic affiliation of their genomes. Interestingly, the dominance of the Betaproteobacteria in freshwater bodies identified in several previous publications [66, 82, 136] was found to be driven by the dominance of the order Burkholderiales [82]. In this context, using amplicon sequencing, Lu et al. [85] reported former Betaproteobacteria as the most dominant class (15.12—46.56%) and *Limnohabitans* (Burkholderiales) at the genus level in freshwater samples from the River Xiangxi. Additionally, critical roles in nutrient and carbon cycling have been related to versatile copiotrophs within the former Betaproteobacteria [50, 15] functions that can be attributed to the dominance of Burkholderiales in freshwater systems [36]. In contrast to the high abundance of Burkholderiales in freshwater, this order is found in relatively low numbers in marine environments, most likely being outcompeted by other Gamma- and Alphaproteobacteria groups.

Dominant species belonging to the order Burkholderiales were similar across all solid substrates and distinct from the planktonic communities (Fig. 1C, Fig. S3 and Table S1B). Amongst them, the most abundant genus corresponded to *Sphaerotilus* (averaging 52, 35, 26 and 0.33% on LDPE, W-LDPE, wood and water, respectively; Fig. S3). *Sphaerotilus* is an aquatic filamentous iron bacterium –a taxon that can use iron as an energy source– also found in activated sludge in WWTP, and capable of forming sheaths that allow attachment to solid surfaces. This favours their growth in slow-running or nutrient-poor water and provides protection by shielding the bacteria from protozoa [81, 139, 98]. Such biofilm-forming bacteria clearly dominate all solid substrates assessed and could potentially serve as the main protective structure for other biofilm colonisers and even organisms that typically have a planktonic lifestyle. For example, the typically planktonic Burkholderiales genus *Limnohabitans* [69] was detected on plastic (LDPE and W-LDPE) and wood (averaging 11, 12 and 13%, respectively), although it was much more abundant in the surrounding water (44%; Fig. 1C). Another interesting Burkholderiales genus identified as part of the biofilms recovered from solid substrates was *Methylotenera*, present across all surfaces tested but more abundant on wood (4.4%) than plastic and water samples (Fig. 1C; 2.2, 1.4, and 0.6% on LDPE, W-LDPE and water, respectively). *Methylotenera* has been described as a putative cellulose degrader found in microbial communities associated with sunken wood logs in marine environments [118]. Similarly, *Duganella* (Burkholderiales) and *Microthrix* (Acidimicrobiales) were found to be significantly more abundant in wood samples (Fig. 1C). Interestingly, *Duganella* was found to encode cellulases, xylan esterases and pectin lyases, all enzymes involved in the degradation of lignocellulosic carbon sources [163]. *Microthrix* species are abundant in active sludges and linked with the degradation of complex carbon compounds [21, 125], however, no direct association with wood or wood derivate degradation has been previously reported. Finally, *Hydrogenophaga* (Burkholderiales was more abundant on solid substrates, especially on W-LDPE (7%; Fig. 1C). Members of this genus have been detected within biofilm-forming bacteria on sand recovered from WWTP denitrification filters [77].

Even though aspects regarding the biodegradation of polyethylene are out of the scope of this investigation, it is important to point out that the genera *Methylotenera* and *Pseudomonas* (Gammaproteobacteria), identified in our samples, have both been associated with the degradation of Polycyclic Aromatic Hydrocarbons (PAHs) in sewage sludge [58] and that several species of *Pseudomonas* have had their degradative capacities widely explored [12, 120, 123]. Curiously, most *Pseudomonas* were significantly more abundant on W-LDPE than in any other sample (Fig. 1C), with the total relative abundance of the family Pseudomonadaceae being 2.6, 18.6, 6.7 and 3.2% on LDPE, W-LDPE, wood and water, respectively (Fig. S3). W-LDPE releases large amounts of organic compounds that encourage the colonisation and growth of a distinct microbial community [48, 159], however, these findings need to be further explored and the degradative capacity of this family elucidated.

At lower taxonomic levels, the water samples showed a clear divergent microbial profile as compared with the solid substrates and contained the typical planktonic genera *Limnohabitans* (41%), *Planktophila* (4.4%), *Polynucleobacter* (4.1%) and *Aquirufa* (0.84%) (Fig. 1C). The most remarkable result from the water samples was the high abundance of potential human pathogens, such as the Enterobacterales *Escherichia*, *Salmonella* and *Klebsiella* (7.2, 3.7 and 1.5%, respectively), as well as *Streptococcus* (3.6%) *–*all described as frequent commensals in waterbodies in the proximity of cities, WWTP, and other industrial activities [6, 72, 80, 122, 124, 131]. As expected from previous studies (e.g. [130], these were also found as part of the microbial community on solid substrates such as plastics, but in much lower abundance (Fig. 1C). The lower abundance of the genus *Escherichia* on solid substrates correlates with previous results [132] where *Escherichia coli* could not be isolated from plastics incubated across different points along the River Weser (Germany). Although most of these potential human pathogens were not abundant on the solid substrates, there were other examples of potential opportunistic human pathogens on these surfaces, such as *Pseudomonas aeruginosa* [43] and *Acinetobacter* [46], which were more abundant in both wood and plastic samples than in water samples (Figs. 1C and S3).

While known biofilm-forming microbes such as *P. aeruginosa* abounded on material surfaces (especially on W-LDPE), Enterobacterales species did not seem to be good colonisers of plastics under *in-situ* environmental settings. Hence, further work is needed to determine whether these potential pathogens colonising plastics may survive, transfer and cause disease [22] and whether they are able to compete with naturally biofilm-forming microbes in freshwater environments.

### ARG distribution within the plastisphere and their surrounding freshwater compartments

Our initial CARD analysis (The Comprehensive Antibiotic Resistance Database) for the identification of ARGs generated a comprehensive list of both known target point mutations for antibiotic resistance (e.g. gyrase and ribosomal mutations) and other antibiotic resistance determinants (i.e. ARGs) (Table S1F). However, we focus hereafter on the latter ARGs due to the elevated background noise that can occur when including point mutation ARGs from metagenomic data. Additionally, the database utilised also provides annotation of genes conferring resistance to disinfectants and other antimicrobial agents; however, as these are not the main target of our research, we will mainly refer to ARGs throughout this investigation.

**Fig. 2:**
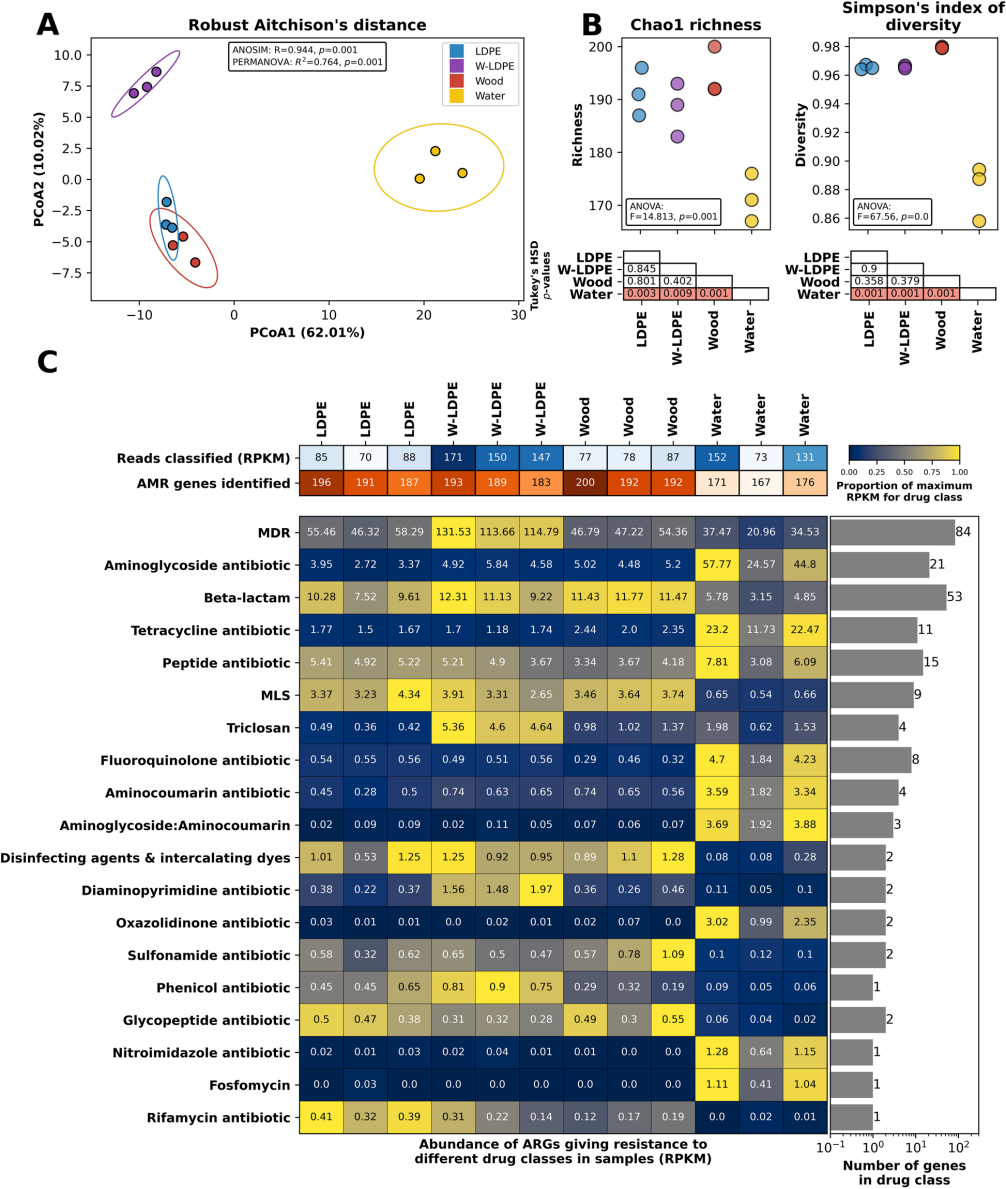
Differential occurrence of disinfectants and antibiotic resistance determinants. Reads within samples were classified using the CARD RGI tool and grouped to the drug class that they gave resistance to. **A** Principal Component Analysis (PCoA) showing samples grouped by Robust Aitchison’s distance (i.e. Euclidean distance of robust Centered-Log Ratio transformed counts). The variation accounted for by each principal component is indicated in parentheses on the axes. Ellipses show the mean plus three standard deviations for each treatment, and the box shows the results of ANOSIM and PERMANOVA tests between the treatments. **B** Chao1 richness or Simpson’s diversity index in each of the three replicates for each treatment (top). The results of ANOVA tests for differences between treatments are shown in boxes within the axes, while *p*-values for *post-hoc* Tukey’s honestly significant difference (HSD) between treatments are shown (highlighted in red are significant values; *p* ≤ 0.05; bottom). Both (**A**) and (**B**) show results for reads classified as ARGs. **C** The number of reads classified (in reads *per* kilobase *per* million, RPKM; blue colour scale) and the number of ARGs identified within each sample (red colour scale). The main heatmap (blue to yellow colour scale) shows the abundance of ARGs giving resistance to different drug classes with the number of genes detected within each drug class shown on the right. Numbers within cells indicate RPKM, while the colour shows the proportion of the maximum for that drug class

As with microbial communities, ARG diversity and distribution showed a noteworthy divergence between the solid substrates and the surrounding water (Fig. 2A and Table S1F). As stated above, taxonomic differences between planktonic communities and biofilms were expected; hence, it is not surprising that these differences in microbial assemblages also drive divergent ARG profiles (Fig. 2A). ARG richness was significantly lower in the planktonic community than in any of the biofilms (ANOVA *p* ≤ 0.05) while the Simpson’s Index of diversity of ARGs was similar between both LDPE substrates and water, with only wood having higher Simpson’s Index of diversity than any other sample type (Fig. 2B). Thus, while biofilms are enriched in ARGs [13, 59], the planktonic microbiome of our *in-situ* incubation site presented an interesting array of ARGs –as demonstrated by our genome-centric analysis below– which is possibly caused by the elevated number of pathogen-like microbes present in the water (e.g. Enterobacterales).

In total, we identified 226 ARG subtypes amongst all microbiomes (212, 209, 211 and 198 identified in the LDPE, W-LDPE, wood and water samples, respectively; Fig. 2C and Table S1F). While the number of ARGs detected kept constant across solid substrates (*n* = 209–212), the relative abundance of the ARGs was clearly higher in W-LDPE biofilms than in any other microbiome (i.e. 156 reads *per* kilobase *per* million [RPKM] in W-LDPE *vs.* 81, 81 and 119 in wood, LDPE and water microbiomes, respectively; Fig. 2C). Multiple Drug Resistance (MDR) genes dominated the dataset in number (*n* = 84) and relative abundance, particularly in surface biofilms (61%, 77% and 66% of the RPKM in wood, W-LDPE and LDPE, respectively*, vs.* 27% in water). MDR is known to dominate ARGs in soil microcosms [31] as well as in mining-impacted soil samples [158], or even in ready-to-eat food [79]. The reported levels of MDR in the literature are in line with our findings, where MDR accounts for a high proportion of the number of reads and the highest number of genes associated with AMR [28, 134, 154] (Fig. 2C).

While MDR, beta-lactams and peptide resistance genes were abundant and similar between biofilm and planktonic microbiomes, other abundantly detected ARG subtypes showed large differences between both microbial communities (Fig. 2C and Table S1F). Hence, planktonic microbiomes were clearly enriched in ARG subtypes for aminoglycosides, tetracycline, aminocoumarin, fluoroquinolones, nitroimidazole, oxazolidinone and fosfomycin; whereas biofilms were enriched in ARGs that conferred resistance to Macrolides-Lincosamides-Streptogramins (MLS), rifamycin, sulfonamides, disinfecting agents and glycopeptides. Interestingly, microbiomes on W-LDPE were specifically enriched for triclosan, phenicol and diaminopyrimidine resistance genes. These results suggest an intrinsic distinctness of ARG profiles within different environmental compartments, mostly driven by microbial community lifestyles, i.e. biofilm *vs.* planktonic, but also influenced by the weathering of plastic surfaces.

**Fig. 3:**
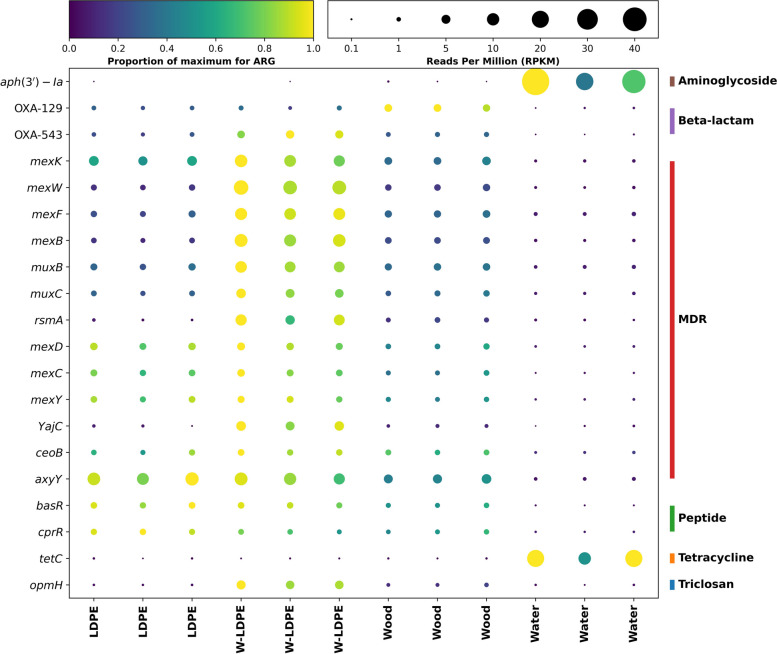
Abundance of the top 20 most abundant ARGs in different samples. The size of the bubble for each ARG (y-axis) represents the abundance in RPKM within each sample (x-axis), while the colour represents the normalised abundance for each gene. The drug class that each ARG gives resistance to is shown on the right

As expected, similar profiles were observed at the individual ARG level (Fig. 3). Thus, water samples were dominated by predominant aminoglycoside resistance gene *aph*(3’)-Ia and tetracycline resistance gene *tet*C. On the other hand, the solid substrates showed high abundances of *axy*Y, *mex* and *mux* genes*,* all belonging to the MDR gene class (Fig. 3). Overall, our results confirm previous studies in which aquatic biofilms –independently of them growing on plastics or natural surfaces– showed high abundances of these MDR genes [134, 154, 157]. Nevertheless, some of these *mex* and *mux* genes, as well as the triclosan resistance genes *opm*H, were substantially more abundant on W-LDPE in our study, emphasising for the first time the concerning enrichment of distinct ARGs on weathered plastics –an observation that requires further attention.

As shown here, plastics have been found to support microbial communities harbouring a variety of resistance genes [35, 144, 157]. The fact that plastic microbiomes are enriched in ARGs has raised wide concern regarding the potential of plastics as reservoirs for antibiotic resistance, although we demonstrate that this is only the case for a distinct set of ARG subtypes and greater attention should be given to all environmental compartments that are impacted by anthropogenic activities since we detected a much higher prevalence of potential pathogens and determined ARG subtypes in the planktonic microbiome. Due to the protection conferred by biofilms and floatability of certain plastics, pathogens and encoded ARGs in planktonic communities may be at a disadvantage when it comes to survival and transport, but are also much harder to filter out and may offer an increased risk of exposure to higher organisms. In this sense, and despite that mutations in antibiotic targets were not considered, fluoroquinolone inactivating determinants –enriched in planktonic microbiomes– confer protection against several second and third-generation drugs, such as ciprofloxacin, levofloxacin and ofloxacin (livertox.nih.gov). It is also worth highlighting that the main concern surrounding the presence of ARGs in the environment is for these to become reservoirs of resistance that can then be horizontally transferred to pathogenic bacteria. Horizontal gene transfer is more likely to occur on solid surfaces, and plastics have been shown to facilitate this process [10], but natural surfaces also need to be taken into account, as demonstrated here. While we chose to perform our *in-situ* incubations downstream from the WWTP effluent because these locations are known for enhancing the abundance of both pathogen-like organisms and ARGs [7, 130], different incubation times, other compartments (e.g. river sediments or WWTP upstream locations), as well as other materials and plastic types, should be contemplated in future studies.

To the best of our knowledge, there are only two other metagenomic datasets that analysed ARGs on plastics incubated in freshwater [101, 154]. Oberbeckmann et al. reported a much higher association of ARGs with wood than with plastics; i.e. 20 putative ARGs conferring resistance to beta-lactams, fluoroquinolones and tetracycline were found in metagenomes from wood samples, while polystyrene (PS) and PE presented only one putative ARG related to beta-lactam resistance on PS and none on PE; [101]. The second metagenomic analysis covered biofilms forming on polyvinyl chloride (PVC) pellets incubated in an *ex-situ* 5 L bioreactor [154]. Researchers determined that even though the biofilms associated with PVC pellets showed some degree of specificity, including a distinct profile of potential pathogens, major differences were only seen when comparing solid substrates *vs.* surrounding water, as reported here in our analysis (Fig. 2). Nevertheless, we further show that plastic weathering prior to water submersion –a process that frequently occurs in nature– enhances the enrichment of particular ARGs. The surface properties of submerged materials are likely to play a key role in the development of microbial community assemblages and, hence, the importance of the choice of adequate control materials for comparison. While we used wood in the present study, more inert materials such as ceramics [115], rocks [154], glass [48] or sand [130] have been previously used as controls. However, these materials may not necessarily exhibit the same physical behaviour in water as plastics (e.g. migration patterns) and, therefore, we selected a buoyant material that would potentially reflect similar effects in terms of dispersal.

### Genome-centric insight of potential pathogens and associated ARGs within the plastisphere

**Fig. 4:**
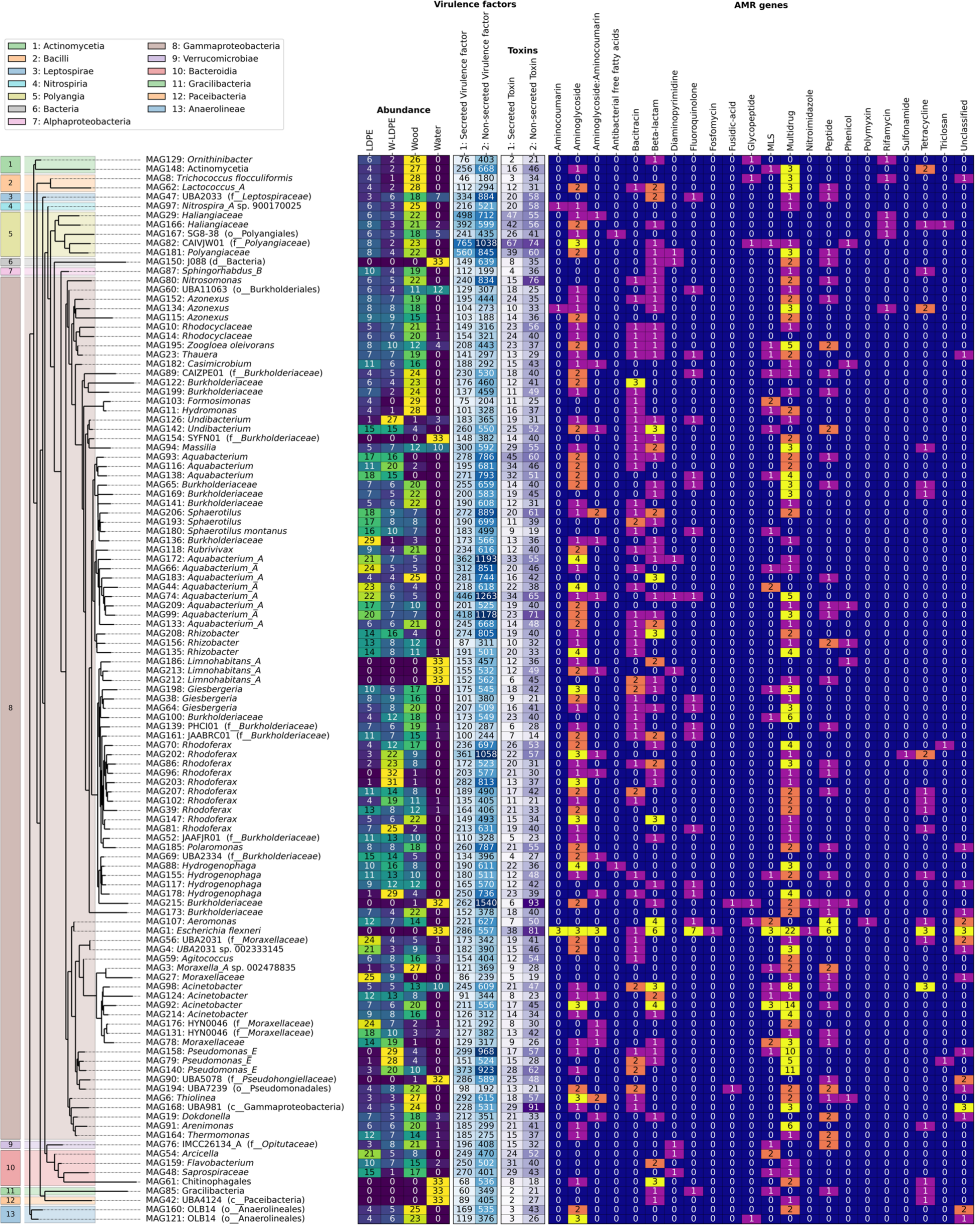
Shortlist of MAGs (*n* = 115) encoding three or more ARGs. MAGs were taxonomically classified with the GTDB toolkit providing the phylogenomic tree shown on the left. The normalised abundance within the different treatments is shown considering triplicate samples. The number of virulence factors, toxins and different ARGs (as predicted by PathoFact) are represented. A summary for all bacterial MAGs (*n* = 214) is available in Fig. S5

In an attempt to link ARGs to their host, we co-assembled the reads from all samples and generated 215 Metagenome Assembled Genomes (MAGs) with > 50% completion and < 10% redundancy (20 of these MAGs were > 90% complete and 73 MAGs were > 75% complete; Table S3A; Fig. S4). Of the 215 MAGs, only one was predicted to be archaeal (MAG106, classified as the TA-21 genus from the Nitrosphaeraceae family; Thermoproteota phylum). The taxonomic classification of the other 214 bacterial MAGs revealed that the contribution of each class closely mirrored that of the read-based analyses: Gammaproteobacteria (113 MAGs), Bacteroidota (45), Myxococcota (11), Alphaproteobacteria (9), Verrucomicrobiota (9) and Actinobacteria (6) (Table S3A). Interestingly, MAGs were assembled for potential pathogens such as *Escherichia flexneri* (MAG1 with 100% completion and 0% redundancy; see *Escherichia/Shigella* reclassification in [109]), *Aeromonas* spp. (MAG107) and *Acinetobacter* spp. (MAGs 98, 124, 92 and 214; Fig. 4). In accordance with our read-based analysis above, *Escherichia* was almost exclusively found in water samples, whereas *Acinetobacter* –typically found in soil and water samples (CDC.gov)– were mainly attached to plastic and wood materials (Fig. 4). While *Acinetobacter* members such as *A. baumannii* are related to pathogenesis in humans [127] (CDC.gov), it was not possible to assign a taxonomic affiliation to the species level for these *Acinetobacter* MAGs. *Aeromonas* spp. are also well-recognised disease-causing agents, not only for animals such as fish, but also for humans [26, 113]

We used PathoFact [39] to predict the ARGs, toxins and virulence factors present within the MAGs and found that of the 214 bacterial MAGs, 115 were predicted to have three or more ARGs in their genomes (Fig. 4). As above, we focussed only on the resistance genes, and not the known target point mutations for antibiotic resistance. Expectedly, MDR genes were the most abundant antibiotic resistance class (i.e. 254 MDR genes within all MAGs, averaging 1.19 MDR genes *per* MAG; Fig. S5). The maximum number of MDR genes predicted for a single genome was 22 in MAG1 (i.e. *Escherichia flexneri*). Other antibiotic resistance classes that were both prevalent and abundant within the MAGs were aminoglycoside and beta-lactam resistance genes, with a total of 93 and 72 (mean 0.715 or 0.5 ARG copies *per* MAG), respectively (Fig. S5). Interestingly, MAG1 (i.e. *E. flexneri*) also had the most beta-lactam resistance gene copies (*n* = 6), as well as three aminoglycoside resistance gene copies. The highest number of aminoglycoside resistance gene copies (*n* = 4) were found in MAGs all belonging to the order Burkholderiales (i.e*.* MAGs 88, 172, 44, and 135; Fig. 4). Genes for bacitracin, MLS or Antimicrobial Peptide resistance were also prevalent, being present in 59, 40 or 49 MAGs, respectively (Fig. S5).

On top of the encoded ARGs, the presence of toxins and virulence factors within the MAGs provides further hints on their potential pathogenicity. Although water samples seem to be the main source of typical human pathogens (e.g*.* MAG1 *E. flexneri*, as well as raw read based detected *Salmonella*, *Streptococcus* and *Klebsiella*, Fig. 1 and Fig. S3), it is important to point out the potential of biofilms –established on either plastic or wood– to also harbour potential opportunistic pathogens (e.g*. Acinetobacter* spp. and *Aeromonas* spp., as well as *P. aeruginosa*, Fig. S3). The array of toxins and virulence factors across most MAGs suggest a wide diversity of pathogenic factors that may affect surrounding organisms; from co-occurring microbes to animal species or plants, as well as humans. For instance, some *Flavobacterium* spp. can cause disease in fish [83]. Regardless of the genomic indications of the potential pathogenicity of the plastisphere (i.e. ARGs and genes encoding virulence factors), it is not possible to draw conclusions on the eventual human risk of plastic pollution as a vector for pathogens without further experimentation. For this, additional assessments are needed to determine the actual pathogenicity of microbes within the plastisphere; these should take into account the potential transfer and ability to cause disease to the host organism –be it human, animal or plant [22].

Altogether, this genome-centric analysis has allowed the assembly of MAG1, i.e*. E. flexneri*, one of many planktonically-found pathogen-like strains detected within our water metagenomes (e.g. *Escherichia*, *Salmonella*, *Klebsiella* and *Streptococcus*; see Fig. 1). Not surprisingly, this MAG showed the highest amount of encoded ARGs and an elevated potential to produce toxins and virulence factors. While these potential pathogens were not major components of the plastisphere, other taxonomical groups like *Acinetobacter* spp. and *Aeromonas* spp. did show a higher presence within the biofilms, in which case, their pathogenic capacity needs to be further elucidated. Since a dominant proportion of environmentally detected ARGs are usually assigned as low-risk or non-clinically relevant [161], it becomes crucial to better assess the actual human health risk posed by plastic pollution in future investigations.

### Case study: sub-inhibitory antibiotics concentrations induce distinct ARG enrichments in different riverine compartments

As our *in-situ* analysis showed a distinct enrichment of ARGs in different riverine compartments, we went on to test the selective pressure sub-inhibitory concentrations of antibiotics may have on the abundance of ARGs in the plastisphere. Antibiotic concentrations considerably below any ecotoxicological effect have been reported to be enough to select for resistances [23]. For this, *ex-situ* microcosms containing river water and sediment were set up with PE, PP and wood fragments (as shown in Fig. S6) in the presence/absence of a cocktail of antibiotics: the macrolide azithromycin (AZM, 0.076 µg L^−1^), the fluoroquinolone ciprofloxacin (CPFX, 0.136 µg L^−1^) and the sulphonamide sulfamethoxazole (SMX, 4.8 µg L^−1^). These are concentrations three orders of magnitude below susceptible breakpoints established by EUCAST (www.eucast.org) and in the range detected in WWTP effluent waters [25]. These clinically relevant antibiotics were selected based on their detection in freshwater environments [149] and because they were previously used in microcosm experiments for ARG selection within microbial communities in the River Sowe [28]. We then used HT-qPCR to achieve an absolute quantification of 48 ARGs -chosen based on the microbial composition and antibiotic resistance profiles revealed by the *in-situ* incubation metagenomic findings. Through this approach, it was possible to obtain a comparative analysis of microbiomes present on PE, PP, wood, water and sediment under varying antibiotic conditions (Resistomap results are shown in Table S4).

**Fig. 5:**
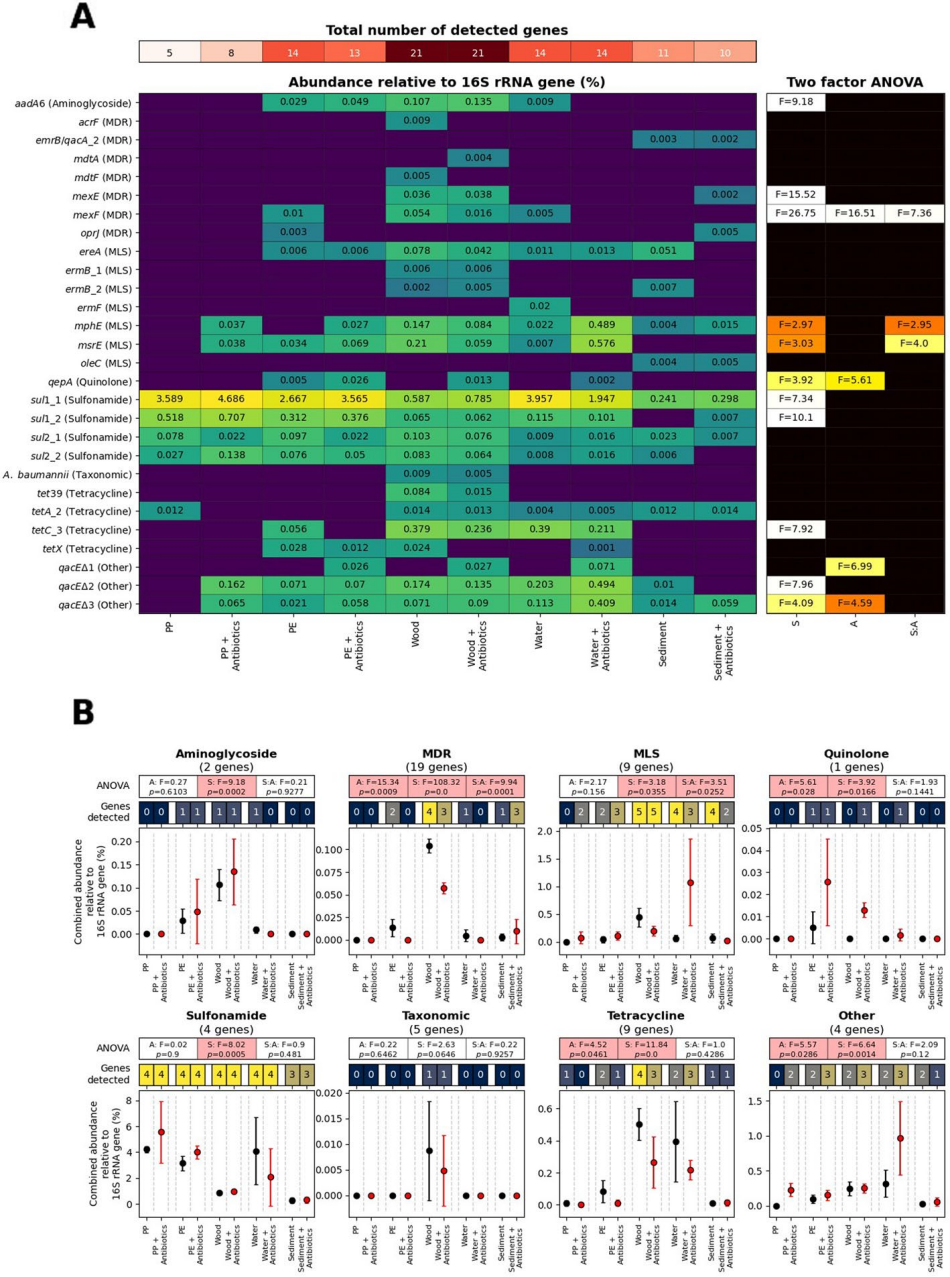
AMR profile of microbial communities in *ex-situ* microcosms exposed to sub-inhibitory antibiotic concentrations and analysed by HT-qPCR. **A** The total number of detected genes in each sample are shown in the red colour scale in the top panel, while the abundance of individual ARGs relative to the 16S rRNA gene is represented in the bottom panel using a blue to yellow colour scale to indicate lowest to highest relative abundances (%) within each row. Dark blue cells represent no detection. The F values for significant (*p* $\le$ 0.05) two-factor ANOVA tests between all samples are also shown with a black-red-white colour scale (right panel). **B** For each drug class, the number of genes tested by Resistomap is shown underneath the title (see Table S4A for full details) together with the ANOVA test on the differences due to the presence of antibiotics (‘A’), surface *vs.* planktonic (‘S’) or both (‘A:S’), with significant results indicated with red shading. ‘Genes detected’ represents the number of the genes within each drug class that were detected *per* sample. The combined abundance of the genes relative to the 16S rRNA gene are graphed, with points indicating the mean and error bars showing the standard deviation

Microbiomes that developed on wood showed the highest detection of ARGs tested (21/48) regardless of the presence/absence of antibiotics (Fig. 5A). Surface biofilms had a significant impact on all antibiotic resistance classes (ANOVA *p* ≤ 0.05), whereas the presence of antibiotics significantly impacted the resistance to quinolones and tetracycline as well as MDR and ‘other’ genes (these being mainly resistance genes against quaternary ammonium compound (QACs); Fig. 5B). Interestingly, while ARGs against quinolones –present within the cocktail as CPFX– showed a positive correlation with the presence of the antibiotic mix, ARGs against tetracycline –antibiotic not present within the cocktail– and MDR genes showed a negative correlation potentially due to environmental selective pressures. Nevertheless, the most remarkable results are observed when analysing the effects at an individual antibiotic and corresponding ARG subtype level. After applying AZM, CPFX and SMX, we would expect an enrichment on ARGs related to MLS, quinolone and sulphonamide resistance, respectively. AZM did in fact cause a strong enrichment of MLS ARGs, particularly of the known resistance genes *msr*E and *mph*E [34], but this occurred mainly in the water samples (Fig. 5A). On the other hand, CPFX enhanced the presence of ARGs against quinolone antibiotics –i.e. gene *qep*A which encodes for a fluoroquinolone efflux pump [156]. The gene *qep*A was enriched in most compartments in the presence of antibiotics, but this was particularly evident in microbiomes from PE and wood surfaces (Fig. 5B). Finally, SMX had an effect on ARGs against sulphonamides. While these genes were particularly high in all conditions, exposure to sub-inhibitory concentrations of SMX enriched for sulphonamide ARGs –i.e. mainly *sul*1– in microbiomes from plastics, PP and PE (Fig. 5). This is particularly interesting as, while previous experiments in our group observed almost negligible effects of such low SMX concentrations in riverine sediments and waters [28], this antibiotic has been shown to adsorb to PE [155] causing the potential enhanced (although not significant) effect of ARG enrichment observed here.

ARGs that confer resistance to antimicrobial compounds not included in the antibiotic cocktail (e.g. aminoglycoside, tetracycline, bacitracin or mechanisms for MDR) showed no observable increase in abundance (Fig. 5 and Table S4). On the other hand, the presence of sub-inhibitory concentrations of antibiotics did, curiously, produce an enrichment of ARGs against antiseptics such as QACs, mainly in the planktonic microbiome (i.e*. qac*E genes classed in the category ‘others’; Fig. 5A). While sub-inhibitory concentrations of QACs are well documented to develop antibiotic resistance in the environment [94, 162], the fact sub-inhibitory concentrations of antibiotics may enrich for QAC resistance –as reported here– has also been previously suggested [95]. This is not surprising given that resistance genes to antibiotics and biocides co-occur in genetic clusters [54, 107]. As performed in Borsetto et al. [28], we also included sediment samples in an effort to better reflect the riverine environment [28]; however, the presence of antibiotics produced no significant variations across all ARGs analysed.

Here we show that sub-inhibitory but environmentally relevant antibiotic concentrations can enhance ARGs in microbiomes from riverine systems. Specifically, our study shows a correlation between the presence of an antibiotic and the enrichment of its particular ARGs, and not a generic non-specific enrichment of ARGs, as well as the co-enrichment of QAC resistance genes. Antibiotic residues have been widely detected in riverine ecosystems [149] and can adsorb to microplastics [155], but distinct ecological compartments seem to be affected by different antibiotics. The influence of an antibiotic on the plastisphere will most likely rely on its adsorption to the plastic’s hydrophobic surfaces or their biofilm penetrability. Hence, this will only occur on a case-by-case basis, opening a new area of investigation that will provide a more detailed view of the potential spread of particular ARGs across the environment using microplastics as vectors.

## Conclusions

Our results indicate that freshwater plastic debris are colonised by potential pathogens that are distinct from those found in their surrounding waters. While emerging pathogenic microbes were enriched on riverine surfaces (e.g. *P. aeruginosa*, *Aeromonas* and *Acinetobacter*), other typical human pathogenic genera (e.g. *Escherichia, Salmonella, Klebsiella and Streptococcus*) were generally restricted to a planktonic lifestyle or outcompeted by other biofilm-forming taxa under environmental conditions. Furthermore, while there were no strong differences between plastic and wood biofilms, weathered LDPE did enrich for certain bacteria such as *P. aeruginosa*, an observation that has not been previously reported and that deserves further investigation. The metagenomics (*in-situ* incubations) and HT-qPCR data (*ex-situ* incubations) have also evidenced the occurrence of a clearly distinct set of ARGs between biofilms and planktonic microbiomes. Of particular note, some biofilm-related ARGs seemed to be more enriched on weathered LDPE than wood or pristine LDPE. To date, the weathering of plastic materials has attracted more attention concerning the increased biodegradability of such recalcitrant polymers [14, 48, 137, 159], and no study has focussed on the differential capacity of weathered plastic to harbour potentially pathogenic bacteria or ARGs compared with pristine materials. Since plastic debris entering the environment is subjected to weathering, it is therefore crucial to examine the impact of materials’ oxidation in relation to their increased capacity to transport potential pathogens.

The exposure to environmentally-relevant concentrations of antibiotics is known to enhance ARGs in environmental microbiomes and, as we show here, plastispheres are no exception. Contrary to what happens in sediments [28], exposure to sub-inhibitory concentrations of antibiotics had a more pronounced influence on the modulation of the ARG profile in biofilms colonising buoyant substrates as well as in their surrounding planktonic microbiome. Nevertheless, only ARGs specific to the antibiotics used were enriched and, furthermore, these differed between compartments, highlighting the specificity of antibiotic diffusion in different microenvironments. These case-by-case variations leave questions open regarding the capacity of plastics to adsorb antibiotics, enrich for specific ARGs and ultimately promote the dissemination of such phenotypes, and how these differ from other environmental compartments.

In this context, given the increase in HT-qPCR and metagenomic studies looking at the presence of clinically relevant pathogens and ARGs on plastics, it is important to stress the urgent need to distinguish between potentially pathogenic taxa (by mere taxonomic association) and phenotypically characterised pathogenic microbial diversity before any claims of plastics serving as vectors for disease-causing microbes are made. Our data highlight the importance of integrating the information from all co-occurring compartments within an anthropogenically-impacted ecosystem and show that the implementation of health and safety measures against the presence of pathogens and ARGs seems to be an issue that goes beyond the plastisphere.

## Methods

### Materials and weathering characterisation of plastic

Commercial packaging films (Greiner Bio-One) of low-density polyethylene (LDPE) were cut into strips of 2 × 10 cm (Fig. S1). Wood sticks were used as a *proxy* of natural control material for microbial colonisation. Additionally, LDPE strips were subjected to thermooxidative weathering (W-LDPE), a process carried out as previously reported [159]. Briefly, LDPE strips were placed in glass beakers and kept in an oven at 80 °C for a period of 6 months at standard atmospheric pressure. The weathering process was monitored by Fourier-transform infrared (FTIR) spectroscopy (PerkinElmer® Spectrum™ GX; [159]. A total of 32 scans were averaged within a range of 600 to 4000 wave cm^−1^ and resolution of 4 cm^−1^ at intervals of 1.0 cm^−1^. The increase of absorbance in the carbonyl peak at 1712 cm^−1^ was used as an indicator of plastic oxidation. The region 2030 cm^−1^ was used as an intensity control since it remains unaltered throughout the oxidation process (Fig. S1) [128].

Square films (4 cm^2^) of pristine LDPE (GoodFellow), PP (GoodFellow) and wood (control material) were used as surfaces for microbial colonisation in the *ex-situ* microcosm incubations.

### River *in-situ* incubations

The weathered and pristine plastic strips, together with the wood sticks, were attached to a PVC frame (Fig. S1). The frame was submerged in the River Sowe, Stoneleigh, UK (52.354944, -1.51425, Fig. S2) for seven days between the 12^th^ and the 19^th^ of February 2020. This location was approximately 1 km downstream from the WWTP Trent Water Ltd. Finham (52.361028, -1.508028; Fig. S2). After the incubation period, samples were collected by cutting triplicate plastic strips and wood sticks off the frame. Samples were placed in sterile square Petri dishes and gently washed three times with sterile river water collected previously from the same location (0.2 µm filtered and further autoclaved at 121 °C for 15 min). Once the samples were washed and loosely attached debris were removed, samples were placed in 50 mL centrifuge tubes (screw cap tubes, Sarstedt, Inc.) containing 20 mL sterile river water and immediately transported for DNA extraction. Additionally, 500 mL of river water was collected into borosilicate bottles (triplicates) using a prefilter mesh of 1 mm (stainless-steel mesh) in order to avoid large fragments. Water was filtered through sterile 0.22 µm membranes (S-pak mixed cellulose esters, 47 mm Ø), and filters were stored at -20 °C until DNA extraction.

### DNA extraction

Samples were sonicated using an ultrasonic bath (Branson 1210) to recover the microbial community attached to the different materials incubated in the river. The ultrasonic bath procedure consisted of 3 rounds of 5 min of sonication with 2 min intervals to avoid overheating and unwanted cell lysis. Immediately after the sonication, detached cells were retrieved by centrifugation (4000 rpm, 15 min, 18 °C). Cell pellets were resuspended using 350 *µ*L of solution MBL and transferred into 2 mL PowerBiofim Bead tubes as the initial step for DNA extraction of the PowerBiofilm Kit (Dneasy® PowerBiofilm®—Qiagen). Fragments of the plastic films and wood were also included in the respective PowerBiofilm Bead tubes. Subsequently, the samples were homogenised by bead-beating (SLS Lab Pro VelociRuptor – Microtube Homogeniser) and further DNA purification was performed as detailed by the manufacturer (Quick-Start Protocol – Dneasy® PowerBiofilm® Kit). The filters resulting from water filtration were also subjected to DNA extraction using the kit as described above. Briefly, the membranes were cut in half and one of the sections transferred into 2 mL PowerBiofilm Bead tubes containing 350 mL of solution MBL. The downstream procedure was the same as described for plastic and wood DNA extractions.

### Metagenomic sequencing and analysis

Shotgun metagenomic analysis was carried out by Novogene (Novogene Europe, Cambridge, UK) using the Illumina NovaSeq 6000 platform (150 bp paired-end strategy). Novogene also performed the library preparation and quality controls. The data output requirement was 20 Gb *per* sample. Biological triplicates of all samples included in this analysis (i.e. LDPE, W-LDPE, wood and water) were sequenced. Kneaddata v0.7.4 [20] was used for primer trimming and quality filtering of reads (using the option “SLIDINGWINDOW:4:20 MINLEN:50”) with Trimmomatic v0.39 [27] and removal of contaminating PhiX sequences (using the option “–very-sensitive –dovetail”) with Bowtie2 v2.3.5.1.

Taxonomic classification was performed using Kraken 2 v2.0.8-beta [151] with confidence thresholds of 0–0.5 at 0.1 intervals (data shown in the main text use a confidence threshold of 0.3) with a database built using all sequences in the NCBI RefSeq release 205 [152]. Bracken v2.5.0 [84] was used to re-estimate abundance and all NCBI taxonomy ID’s for bacteria and archaea were converted into a seven-rank taxonomy [152] using the Genome Taxonomy Database (GTDB, release 207; [108–111, 121]. The NCBI taxonomy was kept for Eukaryotes, Viruses and Archaea. The phylogenetic tree from GTDB (release 207) was used for taxonomic analyses of bacterial reads. ARGs were identified using the Resistance Gene Identifier (RGI) to assign reads to the Comprehensive Antibiotic Resistance Database (CARD) [1] using the Protein Homolog Model and Perfect RGI matches (100% identity). Only completely mapped reads were considered. While the CARD database includes sequences of genes related to resistance against disinfectants and other antimicrobial agents, we will mainly refer to ARGs as they are the primary focus of this investigation.

Metagenome Assembled Genomes (MAGs) were generated with Anv’io v7.0 [47], following the methods of the TARA ocean project [41]. Briefly, MEGAHIT v1.2.9 [78] was used for the co-assembly of paired-end reads from all samples into contigs, which were then filtered to remove any contigs that were below 1000 bp in length. Hidden Markov Models (HMMs) were run to identify single-copy core genes within contigs [63], which were classified taxonomically using Kaiju v1.7.4 [93] with the default database (NCBI non-redundant protein database with the addition of fungi and microbial eukaryotes). Reads from samples were then mapped onto contigs using Bowtie2 v2.3.5.1 [73], generating abundance profiles for each contig across all samples. Contigs that were at least 2500 bp in length were clustered using CONCOCT v1.1.0 [4] to generate 279 bins, 154 of which were above 50% complete. These automatically generated bins were then refined manually to create 215 MAGs that were at least 50% complete with < 10% redundancy.

The quality of MAGs was confirmed using CheckM v1.1.3 [45, 67, 68, 112]. The “complete” PathoFact v1.0 workflow [39] was run using SignalP v5.0b [3] to determine ARG, toxin and virulence factor profiles for each MAG. We applied the default PathoFact parameters for these assignments: 40 for the bitscore threshold of toxin prediction, 0.7 for the plasflow threshold and 1000 bp for the plasflow minimum length. Briefly, PathoFact v1.0 takes assembled contigs and: (1) uses Prodigal (v2.6.3) for prediction of Open Reading Frames; (2) predicts virulence factors using a HMM and a random forest model –both constructed using the Virulence Factor Database [33]– and predicts whether these will be secreted using SignalP,(3) predicts toxins using a HMM –constructed from the Toxin and Toxin Target Database [150]– and predicts whether these will be secreted using SignalP; and (4) predicts ARGs using DeepARG (v1.0.1) [9] and CARD RGI (v5.1.0). We did not use the predictions that PathoFact gave for whether ARGs were found within chromosomes, phages or plasmids, and we manually removed ARGs that were due to mutations or were species-specific genes. We then used v1.7.0 of the GTDB toolkit [32] to obtain the taxonomic affiliation and a phylogenomic tree for all MAGs. The GTDB toolkit also used Prodigal [63] for gene calling, HMMER [45] for marker gene identification, pplacer [89] for inserting genomes into reference trees and FastANI [65] for calculating Average Nucleotide Identity (ANI) and therefore species assignment as well as the additional packages FastTree [119], Mash [106], DendroPy [133], NumPy [60] and tqdm [38].

### Statistical analysis of metagenome samples

Taxa or ARGs with less than 10 reads *per* sample or not present in all three samples of treatment were removed, and data were normalised by conversion to relative abundance. ARGs were normalised to the length of the reference ARG within the CARD database and to the number of reads within each sample to give reads *per* kilobase *per* million (RPKM). Chao1 richness and Simpson’s index of diversity were calculated using the Python package scikit-bio [135]. ANOVA and *post-hoc* Tukey’s HSD tests for differences between groups were carried out using bioinfokit [19]. Robust Aitchison’s distances –euclidean distance on robust Centered Log Ratio (rCLR) transformed abundances using the Python package deicode [88]– between samples were calculated using the Python package SciPy [140], ordinations were performed using the Python package scikit-bio and ANOSIM and PERMANOVA tests between groups were performed using the R package vegan [44]. The Python packages Biopython [37] and ete3 [64] were used for the filtering and rooting of phylogenetic trees, and tree plotting used a modified version of the Python package Biopython.

Following the suggestions of Nearing & Douglas et al. [99], we ran three tools for determining differential abundance: ANCOM-II [87], ALDEx2 [51, 52, 56] and MaAsLin 2 [86]. ANCOM-II and ALDEx2 are both relatively conservative differential abundance methods that identify few false positives; MaAsLin2 is more sensitive than ANCOM-II or ALDEx2 but still controls for false discovery rate [99]. These were run using the R packages exactRankTests, nlme [114], dplyr [148], ggplot2 [147], compositions (van den [138], vegan [44], phyloseq [92], tidyr [146], ALDEx2 and MaAslin 2. Tests were run with: (1) all sample groups, (2) wood *vs.* LDPE, (3) wood *vs.* W-LDPE, and (4) LDPE *vs.* W-LDPE. For a taxon to be considered as significantly differentially abundant, we required a False Discovery Rate cut-off of 0.7 in ANCOM-II, a Benjamini–Hochberg adjusted *p*-value of ≤ 0.1 in ALDEx2 or a q-value of ≤ 0.1 in MaAsLin 2. We report on which of the methods identified a taxon as significantly differentially abundant and considered this to be informative if two of the three methods identified that taxon.

### Microcosms setup

Microcosms were performed in 500 mL glass beakers containing ~ 2 cm of sediment and 350 mL of river water prefiltered through a 1 mm diameter pore mesh to remove large-sized debris. Sediment and water samples were collected in July 2021 from the same location used for our *in-situ* plastic incubations, and microcosms were immediately set up. Square LDPE and PP films as well as wood fragments (4 cm^2^; n = 3 of each material) were added to each microcosm (Fig. S6). Two sets of triplicate microcosms, with and without the addition of antibiotics (azithromycin [AZM, 0.076 µg L^−1^], ciprofloxacin [CPFX, 0.136 µg L^−1^] and sulfamethoxazole [SMX, 4.8 µg L^−1^]), were set up. Microcosms were incubated for 7 days at 20 °C with 40 rpm orbital shaking. After the 7 days, triplicate plastics and wood samples were collected, washed with sterile river water and stored separately in lysis buffer for further DNA extraction as described above. Planktonic microorganisms were collected from 200 mL of the microcosm supernatant water by filtering through 0.22 µm membranes and processed for DNA extraction as detailed above. Sediment samples were collected within the top limit presented in Quick-Start Protocol – Dneasy® PowerBiofilm® Kit (0.2 g of wet sediment material). Downstream DNA extraction was carried out as already described in the DNA extraction section.

### ARG quantification within the *ex-situ* microcosms by HT-qPCR

ARG detection was done using SmartChip™ (Real-Time based HT-qPCR method) performed by Resistomap Oy (Helsinki, Finland). For this procedure, we selected 54 gene targets, which included 48 ARGs and 5 taxon-specific genes as well as the 16S rRNA gene for normalisation (see Table S4). The ARG targets were selected according to our metagenomic analysis's preliminary results. The qPCR conditions were as described previously [141]. The abundance of each ARG was normalised to the 16S rRNA gene in each sample, as calculated by Resistomap Oy (Helsinki, Finland) with a threshold cycle (Ct) of 27 as the detection limit and using the delta Ct calculations previously reported by [97]. To determine differences in the prevalence or abundance of ARGs between the different samples (LDPE, PP, wood, water or sediment) and antibiotic treatments, two-factor ANOVA’s and *post-hoc* Tukey’s HSD tests were carried out using the Python packages statsmodels [129] and bioinfokit [19], respectively.

### Data visualisation

All analyses used custom scripts within R notebooks that used R version 3.6.1, Python version 3.8.10 and the R package reticulate [2]. Basic data importation, exploration and plotting used the Python packages Matplotlib [62], NumPy [60], os, pandas [91] and pickle.

### Supplementary Information

**Additional file 1: Fig. S1.** Materials used for the *in-situ* incubation in the River Sowe. **Fig. S2.** *In-situ *incubation site. **Fig. S3.** Heatmap showing relative abundance at the domain, phylum, class, order, family, genus, and species levels. **Fig. S4.** Phylogenetic tree of the 214 bacterial MAGs generated from the *in-situ* metagenomes. **Fig. S5.** Total and average numbers of ARGs, toxins and virulence factors within all MAGs. **Fig. S6.** Microcosms setup for *ex-situ* experiments exposed to sub-inhibitory concentrations of antibiotics.

**Additional file 2: Table S1.** Metagenomics reads summary, taxonomic annotation, beta diversity, alpha diversity, taxonomic abundance levels and AMR annotation.

**Additional file 3: Table S2.** Results obtained from ANCOM-II, ALDEx2 and MaAsLin2 tools to determine differential abundance of taxa amongst conditions. 

**Additional file 4: Table S3.** MAG summary including AMR, virulence and toxins identified.

**Additional file 5: Table S4.** Resistomap targeted genes and qPCR results.

## Data Availability

Raw sequencing data has been deposited in the European Nucleotide Archive (ENA) under the accession number PRJEB52400 (EMBL-EBI). All commands run and scripts used for analysis can be found at https://github.com/R-Wright-1/river_AMR.
