# Supplementary material for: Microbial hitchhikers harbouring antimicrobial-resistance genes in the riverine plastisphere
Source: Microbiome. 2023 Nov 1;11:225. doi: 10.1186/s40168-023-01662-3 (PMC10619285; doi:10.1186/s40168-023-01662-3)
Supplement: Supplementary file 2 — Additional file 1: Fig. S1. Materials used for the in-situ incubation in the River Sowe. Fig. S2. In-situ incubation site. Fig. S3. Heatmap showing relative abundance at the domain, phylum, class, order, family, genus, and species levels. Fig. S4. Phylogenetic tree of the 214 bacterial MAGs generated from the in-situ metagenomes. Fig. S5. Total and average numbers of ARGs, toxins and virulence factors within all MAGs. Fig. S6. Microcosms setup for ex-situ experiments exposed to sub-inhibitory concentrations of antibiotics. [file 40168_2023_1662_MOESM1_ESM.docx]

**Supplementary Information**

**Microbial hitchhikers harbouring antimicrobial~~-~~resistance genes in the riverine plastisphere**

Vinko Zadjelovic^1^*^T^, Robyn J. Wright^2^, Chiara Borsetto^1^, Jeannelle Quartey^1^, Tyler N. Cairns^1^, Morgan G. I. Langille^2^, Elizabeth M. H. Wellington^1*^, Joseph A. Christie-Oleza^1,3^*

^1^School of Life Sciences, University of Warwick, Coventry CV4 7AL, U.K.

^2^Department of Pharmacology, Faculty of Medicine, Dalhousie University, Halifax, Canada

^3^Department of Biology, University of the Balearic Islands, Palma 07122, Spain.

*Corresponding authors: [vinko.zadjelovic@uantof.cl](mailto:vinko.zadjelovic@uantof.cl), [e.m.h.wellington@warwick.ac.uk](mailto:e.m.h.wellington@warwick.ac.uk) and [joseph.christie@uib.eu](mailto:joseph.christie@uib.eu)

^T^Current address: Centro de Bioinnovación de Antofagasta (CBIA), Facultad de Ciencias del Mar y Recursos Biológicos, Universidad de Antofagasta, Antofagasta 1271155, Chile.

**Fig. S1| Materials used for the *in-situ* incubation in the River Sowe.** (**A**) PVC frame (19×49 cm) to which LDPE, W-LDPE and wood strips were attached. (**B**) FTIR spectra from pristine and weathered LDPE (W-LDPE; thermooxidased material). W-LDPE displays a characteristic carbonyl peak of absorption at 1712 cm^-1^.

**Fig. S2| *In-situ* incubation site.** (**A**) Georeferenced panel grid map of the location of the *in-situ* incubations (**B**) Geographical location of the incubation site in the River Sowe. The wastewater treatment plant (WWTP) located upstream from the incubation site is indicated. (**C**) Characteristics of the incubation site in River Sowe (white star). (**D**) Submerged PVC frame utilised for the incubation of the materials in the river.


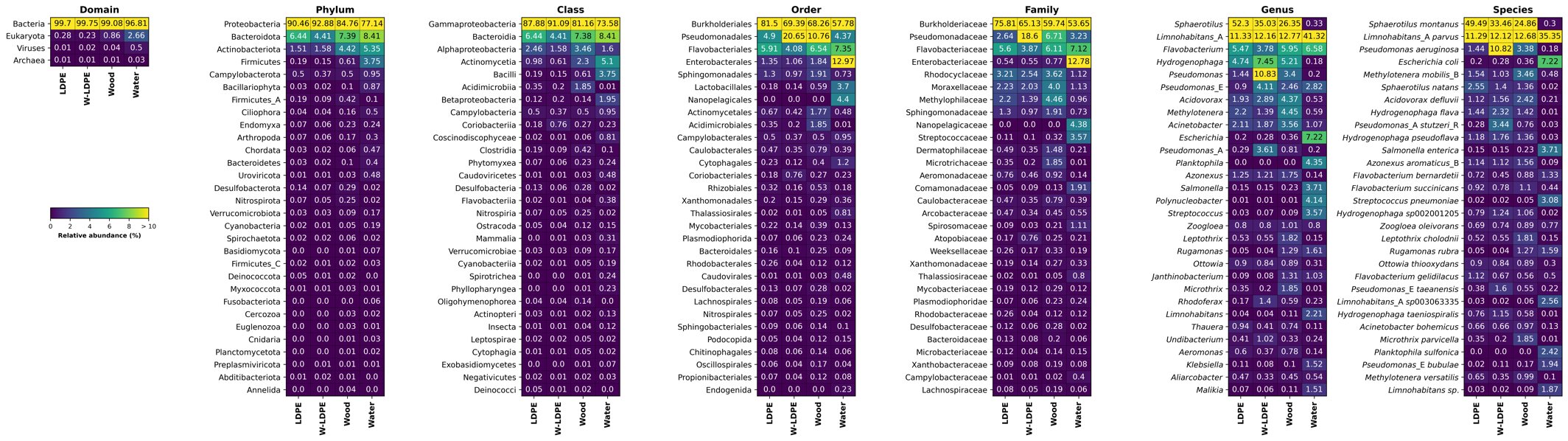


**Fig. S3|** Heatmap showing relative abundance at the domain, phylum, class, order, family, genus, and species levels from left to right, respectively. The mean for three replicates for each sample type is shown, and only the top 30 most abundant taxa are shown for each taxonomic rank, where there were more than 30 present.


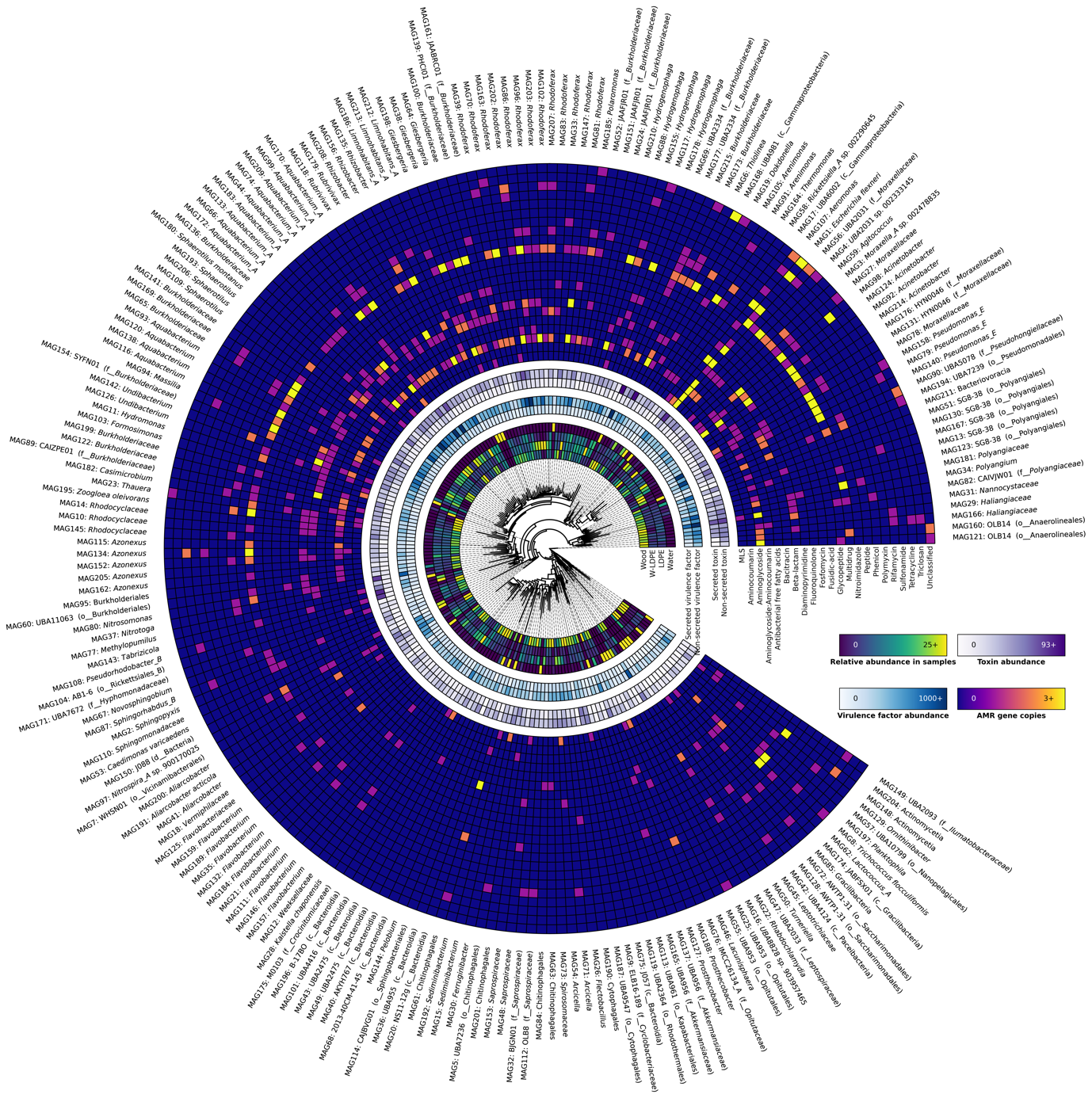


**Fig. S4|** Phylogenetic tree of the 214 bacterial MAGs generated from the *in-situ* metagenomes. Working outwards from the centre: relative abundance of each MAG in each sample (wood, W-LDPE, LDPE and water), secreted and non-secreted virulence factor abundance, secreted and non-secreted toxin abundance and number of ARG copies in each class. The MAG name and GTDB taxonomic classification are shown on the outside.


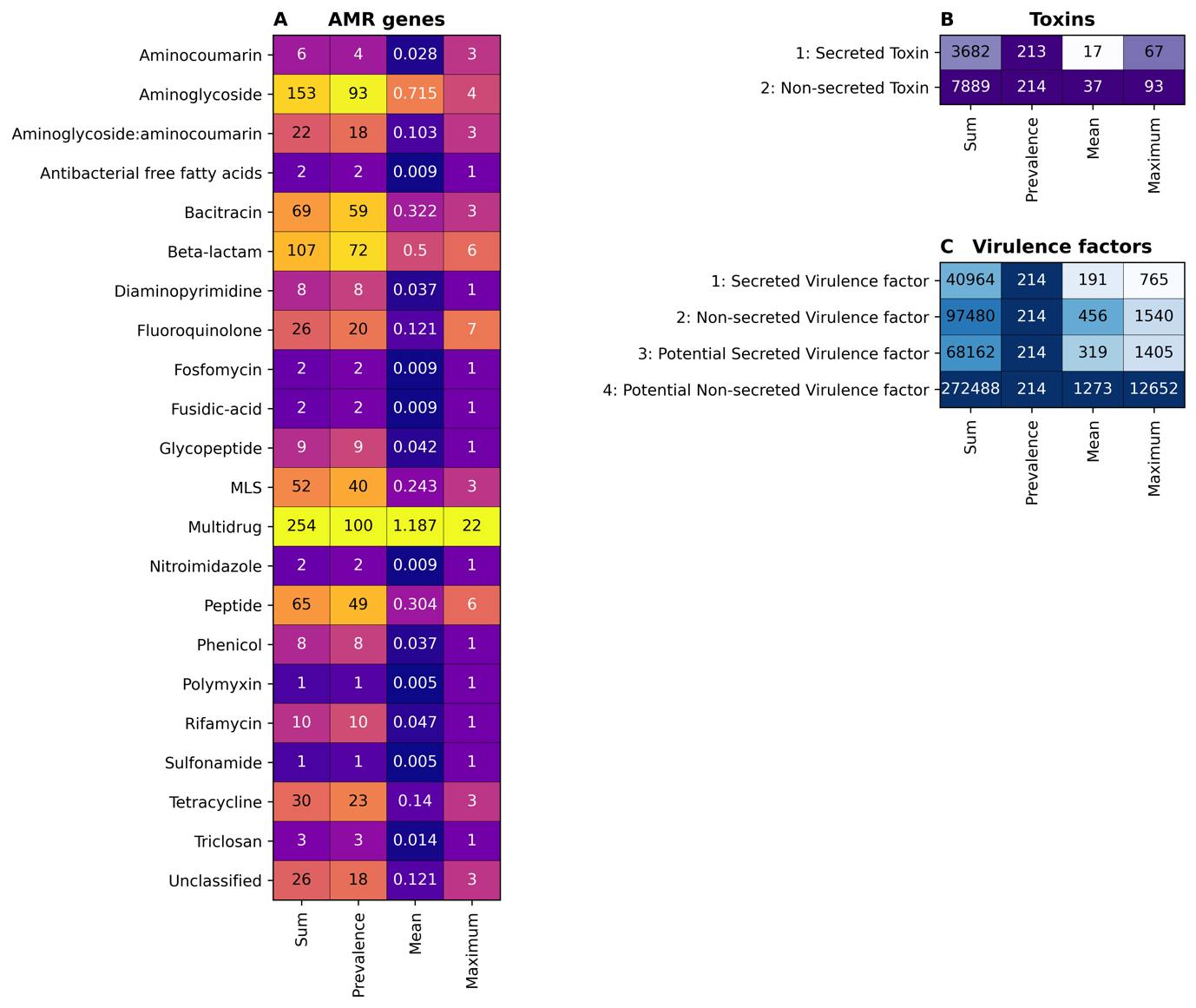


**Fig. S5|** Total and average numbers of (**A**) ARGs, (**B**) toxins and (**C**) virulence factors within all MAGs, as predicted by PathoFact. Each panel shows the sum, prevalence, mean and maximum number of genes giving resistance to each ARG drug class, secreted or non-secreted toxins and (potential) secreted and non-secreted virulence factors, respectively.

**Fig. S6|** Microcosms setup. **A**) Schematic display of microsomes setup including plastic types, controls and the antibiotic included. **B**) Example of microcosms and condition of plastic materials after 7 days of incubation (20°C, 40 rpm).
